# Intestinal Alkaline Phosphatase Deficiency Is Associated with Ischemic Heart Disease

**DOI:** 10.1155/2019/8473565

**Published:** 2019-12-13

**Authors:** Jagannath Malo, Md. Jahangir Alam, Munjareen Shahnaz, Kanakaraju Kaliannan, Gopal Chandra, Tarek Aziz, Tapas Sarker, Mihir Bala, Ratna Paul, Chandan K. Saha, Pradip K. Karmakar, Madhu S. Malo

**Affiliations:** ^1^National Institute of Cardiovascular Diseases, Dhaka 1207, Bangladesh; ^2^Department of Statistics, University of Rajshahi, Rajshahi, Bangladesh; ^3^Bangladesh Medical College, Dhaka 1209, Bangladesh; ^4^Massachusetts General Hospital, Harvard Medical School, Boston, MA 02114, USA; ^5^Dhaka Medical College, Dhaka 1100, Bangladesh; ^6^Shaheed Tajuddin Ahmad Medical College, Gazipur, Dhaka, Bangladesh; ^7^MH Samorita Medical College, Dhaka 1208, Bangladesh; ^8^Diabetic Association of Bangladesh, Dhaka 1000, Bangladesh

## Abstract

**Background:**

We have previously shown that the deficiency of the gut enzyme intestinal alkaline phosphatase (IAP) is associated with type 2 diabetes mellitus (T2DM) in humans, and mice deficient in IAP develop the metabolic syndrome, a precipitant of T2DM and ischemic heart disease (IHD). We hypothesized that IAP deficiency might also be associated with IHD in humans. We aimed to determine the correlation between the IAP level and IHD in humans.

**Methods and Results:**

The IHD patients were recruited from the National Institute of Cardiovascular Diseases (NICVD), Dhaka, Bangladesh, and the control healthy participants were recruited from a suburban community of Dhaka. We determined the IAP level in the stools of 292 IHD patients (187 males, 105 females) and 331 healthy control people (84 males, 247 females). We found that compared to controls, IHD patients have approx. 30% less IAP (mean ± SEM: 63.7 ± 3.5 vs. 44.9 ± 2.1 U/g stool, respectively; *p* < 0.000001), which indicates that IAP deficiency is associated with IHD, and a high level of IAP is probably protective against IHD in humans. The adjusted generalized linear model (GLM) of regression analysis predicted a strong association of IAP with IHD (*p* = 0.0035). Multiple logistic regression analysis showed an independent inverse relationship between the IAP level and the IHD status (odds ratio, OR = 0.993 with 95% CI 0.987-0.998; *p* < 0.01).

**Conclusions:**

IAP deficiency is associated with IHD, and a high level of IAP might be protective against IHD.

## 1. Introduction

The ischemic heart disease (IHD) is the leading cause of death worldwide, making it the major global health problem with devastating consequences in terms of healthcare cost, morbidity, and mortality [1–7]. Globally, approx. 8.9 million people died of IHD in 2015, and more than 400,000 people die annually in the USA from IHD (synonymously known as coronary heart disease (CHD)) [3, 8]. A report from the American Heart Association on IHD showed that an estimated 16.5 million Americans suffer from IHD making the prevalence rate of 6.3% in US adults ≥ 20 years of age [9]. The direct and indirect healthcare cost of IHD in the USA was approx. 177 billion dollars in 2010 [10].

In the context of pathogenesis of IHD, various factors have been implicated, such as hypercholesterolemia, hypertension, diabetes, smoking, genetic predisposition, and depression [11–13]. Pathophysiologically, IHD is a consequence of atherosclerosis, a lipoprotein-driven disease that leads to atheroma (plaque) formation under the arterial endothelial wall through intimal inflammation, necrosis, fibrosis, and calcification [14–16]. Atherosclerotic plaque itself causes obstruction to coronary arterial blood flow precipitating IHD with stable angina pectoris. Acute coronary syndrome (ACS), an acute manifestation of IHD, is nearly always caused by a luminal thrombus or a sudden plaque hemorrhage with or without concomitant vasospasm [17].

Recent advances in basic science established that inflammation plays a pivotal role in coupling dyslipidemia to atheroma formation [17]. In a mouse study, we have demonstrated that endotoxemia, a persistently increased level of bacterial endotoxin lipopolysaccharides (LPS) in the blood, causes low-grade systemic inflammation leading to diabetes and dyslipidemia [18]. Human experimental endotoxemia leads to modest, several-fold increases in the plasma level of cytokines, which closely reflects the subclinical inflammation observed in the metabolic syndrome and chronic cardiovascular diseases [19]. We have shown that the gut enzyme intestinal alkaline phosphatase (IAP) detoxifies LPS, and mice deficient in IAP (Akp3 knockout, Akp3−/−) develop diabetes and dyslipidemia, which could not only be prevented but also reversed by oral supplementation of IAP [18]. Recently, we have shown that IAP deficiency is associated with type 2 diabetes mellitus (T2DM) in humans [20]. Because IAP deficiency results in dyslipidemia in mice, and it is associated with diabetes in humans, we hypothesized that IAP deficiency might also be associated with IHD. Therefore, using a case-control study design, we recruited IHD patients admitted in the National Institute of Cardiovascular Diseases (Dhaka, Bangladesh) and healthy control subjects from a community in the suburb of Dhaka. We determined the level of IAP in the stools of these participants, and we found that IAP deficiency is strongly associated with IHD. This suggests that “temporal IAP profiling” might be a valuable tool for identifying IAP deficiency and thus diagnosing healthy people with “incipient (latent) IHD” in case of probable IAP deficiency-mediated pathogenesis of IHD.

## 2. Methods

### 2.1. Study Design and Participants

With the aim to define a possible association of IAP deficiency with ischemic heart disease (IHD), a case-control study of patients with IHD and control healthy participants was designed to determine the difference between two groups in the context of stool IAP concentrations (case: IHD; control: healthy). The IHD group, aged 35-70 yr., included 292 participants (187 males and 105 females) who were recruited from the patients admitted in the National Institute of Cardiovascular Diseases (NICVD), Dhaka, Bangladesh. The control healthy participants (total 331: 84 males and 247 females), also aged 35-70 yr., were recruited from a suburban community of Dhaka by advertisement through local dignitaries, hospitals, clinics, and physicians' offices as described in detail previously [20]. Based on unpublished preliminary data, the sample size of each group was determined to accomplish statistical power of 80% or more (continuous endpoint, *α* = 0.05). The IHD patients had a history of stable or unstable angina pectoris, and myocardial infarction (MI) with or without ST-segment elevation in electrocardiographs (EKG). The diagnosis was confirmed by coronary angiography. Most of the patients in the study had significant coronary artery disease and were treated by angioplasty and stenting, and antihypertension and antidyslipidemia (anticholesterol) medications. Any person suffering from an acute disease was excluded from the study as described in detail previously [20]. Briefly, participants with a history of type 1 diabetes, type 2 diabetes, and cancer were excluded. Pregnant women were also excluded. Hepatic and renal functions of each participant were evaluated by physical and biochemical tests, and participants with significant hepatic and renal diseases were excluded. Any participant with a history of chronic alcohol consumption was also excluded.

The study protocol was reviewed and approved by the Bangladesh Medical Research Council (registration number: 150 02 10 2018, Ref.: BMRC/NREC/2016-2019/28). Each participant signed an informed consent form to participate in the study.

### 2.2. Study Limitations

The control participants were recruited from a suburban community of Dhaka, Bangladesh, where most of the male population leave home early in the morning for their jobs making it difficult to collect fasting blood sample from male participants, and as a result, the sample size of male control participants was limited. However, the sample size was adequate to achieve >80% statistical power for the study.

### 2.3. Laboratory Tests, Physical Examination, and Sociomedical History

The protocols for physical examination, sociomedical history, and laboratory tests have been described in detail previously [20]. Biochemical assays were performed using kits from Linear Chemicals S.L. (Barcelona, Spain) and an automatic chemistry analyzer from Sinnowa Medical Science & Technology Co., Ltd (Nanjing, Jiangsu, China; model: Sinnolab MT 5000, Version 5.00). All participants were subjected to overnight fasting (10 h) followed by biochemical tests for plasma glucose, and serum cholesterol, triglycerides, low-density lipoproteins (LDL), high-density lipoprotein (HDL), alanine aminotransferase (ALT), and creatinine. Physical examination of a participant included measuring height, weight, temperature, and blood pressure. BMI was calculated as weight in kg divided by the square of height in meter (kg/m^2^). Participants were asked for the history of diabetes, heart, kidney, and liver diseases, and also alcohol consumption.

### 2.4. Homogenization of Stool

Alkaline phosphatase (AP) assay was performed on the supernatant of a homogenized stool suspension as previously described [20]. Briefly, a small amount of fresh stool (mgs) was measured and then suspended in the “stool dilution buffer” (10 mM Tris-HCl, pH 8.0, 1 mM magnesium chloride, 10 *μ*M zinc chloride) at a defined ratio (1 mg stool: 50 *μ*l of stool dilution buffer). A homogenous stool suspension was prepared by vigorous vortexing. The sample was then centrifuged at 10,000 x g for 20 min, and the supernatant containing AP was used for measuring AP concentration.

### 2.5. Alkaline Phosphatase Assay

Alkaline phosphatase (AP) in the stool supernatant was assayed following an established protocol using the automatic biochemistry analyzer mentioned above (Nanjing, Jiangsu, China) as previously described [20]. Briefly, 20 *μ*l of supernatant was mixed with 1 ml of enzyme assay buffer (1.25 M diethanolamine (DEA) buffer, pH 10.2, 0.6 mM magnesium chloride) containing 10 mM p-nitrophenyl phosphate (pNPP). The reaction mixture was incubated for one min at 37°C, and then, AP concentration was measured by the analyzer precalibrated with AP standards. Because most of the AP activity in stool is due to IAP, the stool AP values are expressed as units of IAP/g stool as previously defined [20]. A single laboratory technologist who was blinded to the diagnoses of participants performed all the AP assays.

### 2.6. Statistical Analysis

Statistical analysis was performed using the SAS 9.4 System (SAS Institute, Inc., Cary, North Carolina) as previously described [20]. Mean and standard errors were determined for controls and IHD cases stratified by gender. The correlation between IAP concentrations and various risk factors for IHD was evaluated by Pearson correlation coefficients stratified by gender and IHD status (IHD patients or non-IHD controls). Mean difference in the IAP level between IHD cases and non-IHD controls was calculated by the general linear model (GLM) of regression analysis controlling for the effects of sex, age, BMI, creatinine, cholesterol, HDL, LDL, triglycerides, ALT, FPG, systolic blood pressure, and diastolic blood pressure on the IAP level and IHD status. The statistical significance of the variance associated with independent variables was determined from sum of square III using the GLM procedure in SAS. Multiple logistic regression analysis was performed using PROC LOGISTIC procedure in SAS, and the association between IHD cases and independent risk factors including IAP was determined. The independent risk contribution of IAP to IHD status was evaluated based on regression coefficients and odds ratio. Unpaired two-tailed Student's *t*-test or Wilcoxon two independent sample test (Mann–Whitney *U* test) was used to calculate the statistical significance of the difference between two groups. The difference between two groups was considered statistically significant when the *p* value was <0.05. An online program was used to perform post hoc statistical power analysis of two independent groups (http://clincalc.com/Stats/Power.aspx).

## 3. Results

### 3.1. Patients with Ischemic Heart Disease (IHD) Have Low Levels of IAP in Their Stools

We determined the levels of IAP in the stools of 292 IHD patients (187 males and 105 females) and 331 healthy controls (84 males and 247 females). Participants were 35-70 yr. old, and there was no significant difference in age between respective control and IHD groups.  Table 1 provides the average value (mean ± SEM) of a few important physical and biochemical characteristics of participants, such as age, weight, height, body mass index (BMI), systolic and diastolic blood pressures, and levels of serum creatinine, cholesterol, HDL-cholesterol, LDL-cholesterol, triglycerides, alanine aminotransferase (ALT), and fasting plasma glucose (FPG).  Table 2 describes the median (range) value of physical and biochemical characteristics of participants.

**Figure 1:**
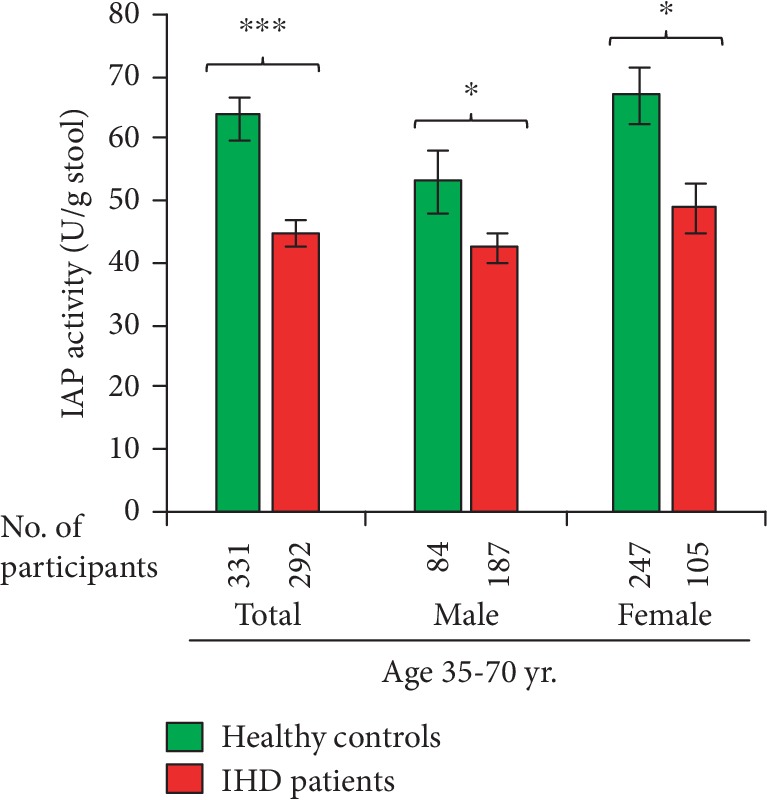
Patients with ischemic heart disease (IHD) have low level of intestinal alkaline phosphatase (IAP) in their stool. Stool samples of control healthy participants and IHD patients were homogenized in a stool dilution buffer followed by centrifugation and collection of supernatant. The supernatant was assayed for IAP concentration using an automatic biochemistry analyzer. *Statistics*: values are expressed as mean ± SEM. Statistical significance of the difference between two groups was tested using the unpaired two-tailed Student's *t*-test. *p* < 0.05 is considered significant. ^∗^*p* < 0.05, ^∗∗∗^*p* < 0.001. The post hoc statistical power of the study was 99.6%, validating the adequacy of power (conventionally, >80% power at *α* = 0.05) for sample sizes. Percentage of IAP in IHD patients compared to healthy controls: total, 70.5%; male, 79.9%; female, 72.9%. The average IAP level is 20.7% less in healthy control males compared to healthy females; however, the difference is not significant (*p* = 0.087223). The average IAP level is 13.0% less in IHD males compared to IHD females; however, the difference is not significant (*p* = 0.140301).

IAP concentrations in the stools of entire healthy control and IHD populations are shown in Figure 1. Compared to healthy controls, IHD patients have approximately 29.5% less IAP (mean ± SEM: 63.8 ± 3.5 vs. 44.9 ± 2.1 U/g stool, respectively; *p* = 0.000001; median (range): 44.51 (1.97-389.72) vs. 36.12 (1.90-187.78) U/g stool, respectively). Also, sex-dependent distributions show similar reduction in IAP levels in male and female IHD patients. Compared to control healthy males, IHD male patients have 20.1% less IAP (53.6 ± 5.4 vs. 42.7 ± 2.3 U/g stool, respectively; *p* = 0.032118; median (range): 36.09 (3.10-203.98) vs. 34.29 (2.21-118.37) U/g stool, respectively). Similarly, in comparison to healthy females, IHD females have 27.1% less IAP (67.7 ± 4.4 vs. 49.4 ± 4.0 U/g stool, respectively; *p* = 0.011342; median (range): 47.43 (1.97-389.72) vs. 39.22 (1.90-187.78) U/g stool, respectively). Healthy control males have 20.8% less IAP compared to healthy females; however, the difference is not significant (*p* = 0.087223). Similarly, the IHD males have 13.0% lower IAP level compared to IHD females, and the difference is not statistically significant (*p* = 0.140301). The above data indicate that IAP deficiency is associated with IHD, and a high level of IAP probably plays a protective role against IHD.

### 3.2. IAP Levels Are Low in IHD Patients at Most Percentile Points

**Figure 2:**
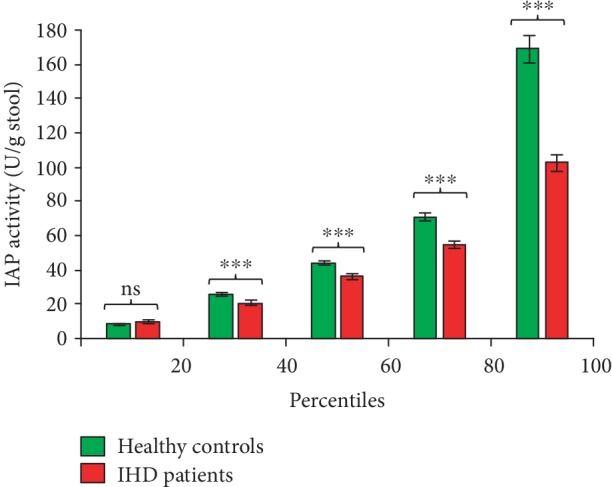
IAP levels are low in IHD patients at most percentile points. Individual IAP values from each group (healthy controls or IHD patients) were arranged from the lowest to the highest, and then, the average IAP value within each 20th percentile was calculated (*n* = 29 within each 20th percentile for IHD patients, and *n* = 66 within each 20th percentile for healthy controls). Average values for corresponding percentiles are plotted. Statistics: values are expressed as mean ± SEM. Statistical significance of the difference between two respective groups was tested using the unpaired two-tailed Student's *t*-test. *p* < 0.05 is considered significant. ns: not significant; ^∗∗∗^*p* < 0.001. Note: only the values in first and last 20 percentile divisions will be greatly affected if an “outlier” (a few extremely high or low values, compared to the most other values, affecting the mean value) is present. The values within 20th and 80th percentiles are real, not affected by outliers.

Outliers, the extremely high or low values compared to the most other values, affect the average (mean) value. Taking in consideration of any influence of outliers on any average value (see Figure 1), we further verified the difference in IAP levels between IHD and healthy control groups at different percentile points. We evaluated the percentile distribution of IAP values in 292 IHD patients and 331 healthy controls. We organized individual IAP values from each group (IHD and control) from the lowest to the highest and then calculated the average IAP value within each 20 percentile divisions. It is evident that, except within first 20 percentiles, at all other percentile points, IAP values are less in IHD patients compared to the healthy population, and the difference is highly significant (Figure 2). The percentile distribution of IAP levels confirms that IHD patients, indeed, have lower levels of IAP compared to their counterparts. This distribution further predicts that IAP probably plays a protective role against IHD.

### 3.3. IAP Deficiency Is Associated with IHD

We determined Pearson correlation coefficients (Table 3) and found no correlation between the IAP level and age, gender, FPG, BMI, ALT, serum creatinine, blood pressure, and lipid parameters that confirmed our previous observation [20]. A generalized linear regression model predicted a strong association of IAP with IHD (Table 4). Multiple logistic regression analysis controlling for age, gender, FPG, BMI, ALT, serum creatinine, blood pressure, and lipid parameters showed a significant independent inverse relationship between the IAP level and the log-odds of IHD (Table 5, regression coefficient = −0.00730, *p* < 0.01). For one unit (U/g stool) increase in the IAP level, the odds of developing IHD decreases by 1% controlling for other risk factors (OR = 0.993 with 95% CI 0.987-0.998, *p* < 0.01). This indicates that a high IAP level might be protective against IHD. Our fitted logistic regression model has a very good predictive power (area under the curve, AUC = 0.89).

### 3.4. Obese Women with High IAP Do Not Develop IHD

**Figure 3:**
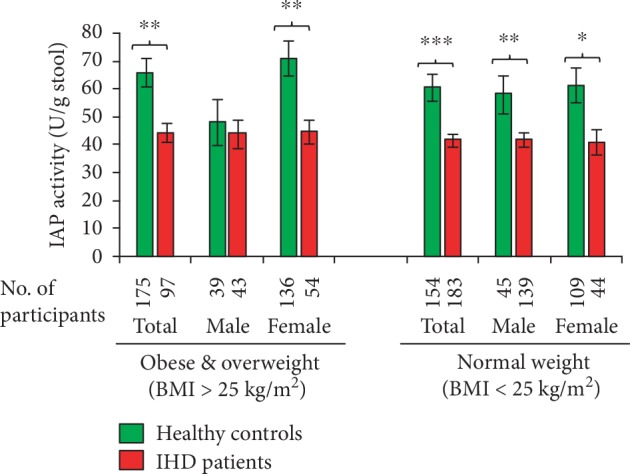
Obese women with high IAP do not develop IHD. Stool samples of healthy participants and IHD patients were assayed for IAP concentration using an automatic biochemistry analyzer (see Figure 1). The healthy controls as well as IHD patients were categorized in two groups, one with high BMI (>25.0 kg/m^2^) and the other having low BMI (<25.0 kg/m^2^). *Statistics*: values are expressed as mean ± SEM. Statistical significance of the difference between two groups was tested using the unpaired two-tailed Student's *t*-test. *p* < 0.05 is considered significant. ^∗^*p* < 0.05; ^∗∗^*p* < 0.01; ^∗∗∗^*p* < 0.001. Note: the IAP values are mildly higher in obese male control participants compared to obese male IHD patients; however, the difference is not significant (*p* = 0.648285). There is no significant difference in IAP levels between high- and low-BMI groups of healthy controls as well as between high- and low-BMI groups of IHD patients. Also, there is no significant difference in IAP levels between males and females of high- and low-BMI groups of the respective study population.

Obesity (BMI > 30.0 kg/m^2^) as well as overweight (BMI > 25.0 kg/m^2^) has been recognized as a risk factor for IHD [16, 21]. We categorized the healthy controls and IHD patients of this study in two groups, one with BMI > 25.0 kg/m^2^ and the other having BMI < 25.0 kg/m^2^. IAP levels in high- and low-BMI groups are shown in Figure 3. It is evident that in case of total populations both groups of IHD participants have significantly lower amounts of IAP compared to their healthy counterparts. It is also obvious that an obese or nonobese woman with high IAP belongs to the healthy control group, but not to the IHD group. Nonobese healthy control males have significantly higher level of IAP compared to nonobese IHD males; however, obese healthy men have only insignificantly higher level of IAP compared to obese male IHD patients. These data suggest that a high level of IAP probably plays a protective role in women against the development of IHD irrespective of obesity. On the other hand, obesity might repress IAP in males, consequently leading to IHD.

## 4. Discussion

Ischemic heart disease (IHD) is the number one cause of death in the world, and IHD is preceded by the metabolic syndrome consisting of obesity, hyperglycemia, hypertension, hypertriglyceridemia, and low high-density lipoprotein (HDL) cholesterol [20–22]. We have previously shown that mice deficient in the gut enzyme intestinal alkaline phosphatase (IAP) develop the metabolic syndrome that ultimately leads to diabetes and dyslipidemia (hypertriglyceridemia, hypercholesterolemia, low HDL-cholesterol, high low-density lipoprotein (LDL) cholesterol) [18]. Based on these mouse data, we hypothesized that IAP deficiency might be associated with diabetes and IHD in humans. Indeed, we have recently shown that type 2 diabetes mellitus (T2DM) is associated with IAP deficiency establishing a possible protective role of IAP against T2DM [20]. In this current study, we determined the level of IAP in IHD patients, and as expected, we observed that similar to T2DM, IHD is also associated with IAP deficiency. We found that compared to healthy control participants, patients with IHD have approx. 30% less IAP that indicates an apparent protective role of IAP against IHD, which is in concordance with the observation that a high level of IAP is protective against dyslipidemia in mice [18]. Pearson correlation coefficients (Table 3) confirmed our previous observation that there is no correlation between the IAP level and age, gender, FPG, BMI, ALT, serum creatinine, blood pressure, and lipid parameters [20]. Generalized linear modeling (GLM) and multiple logistic regression analyses revealed an independent association between the IAP level and the IHD status (see Tables 4 and 5). Obesity has been postulated to be a risk factor for IHD [16, 21]; however, we discovered a possible protective role of high IAP against IHD irrespective of obesity in women (see Figure 3). The study showed insignificant difference in IAP levels of obese healthy men and obese IHD men (see Figure 3). This observation indicates that, besides other repressors of IAP, probably obesity also represses IAP in men; however, such obesity-mediated repression of IAP is possibly counteracted in females by female sex hormones.

We found that approx. 23.3% IHD patients have IAP level > 65.0 U/g stool (the average value of healthy controls), and based on this observation, we speculate that either IAP is not associated with the pathogenesis of IHD in these specific patients or a persistent loss of IAP from a previously higher level might also lead to IHD. We also speculate that approx. 30% loss of IAP activity might be significant to precipitate IHD as IHD patients on average have approx. 30% less IAP compared to their healthy counterparts (see Figure 1). Further, it appears that compared to men, women will probably be able to tolerate more loss of IAP (see Figure 1) in the context of any likely IAP deficiency-mediated pathogenesis of IHD.

We have previously discussed in details on the confounding factors modulating the alkaline phosphatase (AP) level in stool [20]. In brief, we observed that approx. 80% stool AP activity is due to IAP, and the rest AP activity is mostly due to bacterial AP as the human tissue nonspecific AP (TNAP) in stool is very low. Because most of the stool AP activity is due to IAP, we referred stool AP as IAP in the previous report as well as in this report. It has been reported that the IAP level can be modulated by some dietary components, such as curcumin, omega-3 fatty acid, and alcohol [20, 23, 24]. However, we believe it is very unlikely that diets had any significant effect on these data because the sample sizes were relatively large (achieved >80% statistical power) and participants were on unrestricted diets.

Previously, we have shown that IAP deficiency in mice leads to the development of the metabolic syndrome, the precipitant of diabetes and IHD [18], and IAP deficiency is also associated with diabetes in humans [20]. In our previous study [20], we observed that approx. 65% of healthy persons have less than average level of IAP (65 U/g stool), and we hypothesized that these people have “incipient (latent) metabolic syndrome” including incipient diabetes and incipient IHD and are very vulnerable to develop diabetes, IHD, and other metabolic diseases. We strongly believe that IAP deficiency causes diabetes as well as IHD in humans, and to confirm this hypothesis, we anticipate that it will require a long-term prospective cohort study regularly monitoring stool IAP and incidence rates of diabetes and IHD in a group of healthy people having “IAP deficiency (defined as IAP conc.<65 U/g stool)” and another group of healthy people having “no IAP deficiency (defined as IAP conc.>65 U/g stool).” Provided IAP deficiency causes diabetes and IHD, we envision that regular monitoring of stool IAP (alternatively, SAP, stool alkaline phosphatase), defined as “temporal IAP profiling,” will be a very valuable tool in diagnosing incipient diabetes and IHD. We think that childhood obesity and associated metabolic syndrome [25] is possibly due to IAP deficiency and should be investigated.

We have previously shown that IAP oral supplementation prevents diabetes and dyslipidemia in mice [18]. In the context of IAP deficiency in diabetes, we have discussed a probable use of oral IAP supplementation for the prevention of diabetes [20]. Similar to the association of IAP deficiency with diabetes, here, we report an association of IAP deficiency with IHD. Accordingly, we believe that oral IAP supplementation could also be a therapeutic approach to prevent IHD provided that a causal role of IAP deficiency in the pathogenesis of IHD is well established by a longitudinal cohort study as mentioned above. As previously discussed, IAP could also be upregulated by small molecules, such as short-chain fatty acids (as sodium butyrate and propionate), curcumin, omega-3 fatty acid, and thyroid hormone [20, 26–28]. We believe that defining the mechanism of IAP deficiency will be pivotal to understand the pathophysiology of IHD, diabetes, and other metabolic diseases if IAP deficiency is really involved in the pathogenesis of these diseases. It is postulated that dysbiosis leads to the metabolic syndrome [18, 20, 29, 30], and therefore, it is anticipated that dysbiosis might play a role in IAP deficiency. We believe that genetic, environmental, and/or nutritional factors also might be involved in IAP deficiency and need to be investigated in the future.

In the context of molecular mechanism of how IAP deficiency might lead to the development of IHD, we hypothesize a pathway that IAP deficiency precipitates chronic endotoxemia followed by chronic systemic inflammation that subsequently leads to IHD (IAP deficiency→chronic endotoxemia→chronic systemic inflammation→vascular endothelial tissue damage→IHD). IAP detoxifies LPS, and accordingly, in support of our hypothesis, we propose that IAP deficiency causes an increase in the intraluminal endotoxin LPS concentration that might lead to excess LPS translocation to systemic circulation thus precipitating endotoxemia. Also, we have shown that IAP deficiency causes increased intestinal permeability which, we believe, leads to translocation of LPS to the systemic circulation thus causing endotoxemia [18]. Furthermore, it is known that LPS binds to fatty acid, and we have shown that wild-type mice on a high-fat diet develop endotoxemia that could be prevented by oral IAP supplementation [18]. It is apparent that IAP deficiency could lead to endotoxemia due to (1) increased accumulation of LPS in the gut, (2) increased gut permeability, and (3) failure of high-fat diet-mediated endotoxemia. We have previously reported that IAP deficiency-induced endotoxemia leads to systemic inflammation as evidenced by increased serum TNF-*α* [18]. It has been well documented that chronic systemic inflammation causes damage to vascular endothelial cells leading to atherosclerosis and coronary artery disease [18, 31]. Also, we have shown that IAP-deficient mice develop dyslipidemia, the precipitant of atherosclerosis and coronary artery disease [18].

Intestinal alkaline phosphatase is a membrane-bound glycoprotein that optimally functions at a high pH. Functions and regulations of IAP have been extensively reviewed [24, 25, 32, 33]. IAP is exclusively expressed in villus-associated enterocytes of proximal small intestine and hence recognized as an enterocyte differentiation marker [25, 34, 35]. IAP is secreted from villus-associated enterocytes, and most amount of the enzyme moves into the intestinal lumen, whereas a small amount of IAP goes to blood circulation [36]. IAP that is secreted into the intestinal lumen moves downwards from the proximal small intestine to the distal large intestine and then excreted with stool [37].

Physiologically, IAP exerts a few critical functions that are fundamentally important for the propagation of life. It is an anti-inflammatory factor that detoxifies different inflammatory agents, such as bacterial endotoxin lipopolysaccharides (LPS) and lipoteichoic acids (LTA), CpG DNA, flagellin, and uridine diphosphate (UDP), wherein IAP detoxifies these targets by dephosphorylation (phosphohydrolysis) [38–41]. Another critical function of IAP is to maintain the intestinal microbiotal homeostasis and prevention of pathogenic bacterial infection [37]. In the context of microbiotal homeostasis, IAP promotes the gut bacterial growth by reducing the concentrations of intestinal luminal nucleotide triphosphates (ATP, dATP, etc.) that have toxic effect on bacterial growth [42]. Also, IAP limits fat absorption and maintains the gut mucosal integrity [43, 44].

Regarding the pharmaceutical use of IAP, we have shown in a mouse model that oral IAP supplementation prevents antibiotic-induced susceptibility to enteric pathogens such as *Clostridium difficile* and *Salmonella typhimurium* [37, 45]. We have also shown that IAP supplementation prevents diabetes and dyslipidemia in mice [18]. Furthermore, IAP supplementation has been shown to have therapeutic value in treating colitis, acute kidney injury, and peritonitis in humans and animal models [46–51].

## 5. Conclusions

In conclusion, this study establishes that IAP deficiency in stool is associated with IHD, and a high level of IAP might play a protective role against IHD. Furthermore, the study suggests a role of IAP as a biomarker for diagnosing any possible IAP deficiency-mediated pathogenesis of “the incipient metabolic syndrome” including “incipient IHD.”

## Figures and Tables

**Table 1:** Characteristics of healthy control participants and ischemic heart disease (IHD) patients.

Characteristics	Total participants		Males		Females	
	Healthy	IHD	Healthy	IHD	Healthy	IHD
No. of participants	331	292	84	187	247	105
Age of participants (yr.)	50.8 ± 0.4	52.4 ± 0.5^∗^	52.3 ± 0.7	53.4 ± 0.7	50.3 ± 0.5	50.6 ± 0.9
Weight (kg)	58.2 ± 0.6	60.8±0.6^∗∗^	64.6 ± 1.1	63.0 ± 0.7	56.0 ± 0.7	56.8 ± 0.8
Height (m)	1.5 ± 0.01	1.6±0.01^∗∗∗^	1.6 ± 0.01	1.6 ± 0.00^∗^	1.5 ± 0.00	1.5 ± 0.01
BMI (kg/m^2^)	25.5 ± 0.2	24.1 ± 0.3	23.2 ± 0.3	24.1 ± 0.1^∗^	25.7 ± 0.3	25.7 ± 0.4
Systolic blood pressure (mmHg)^a^	136.2 ± 1.3	119.7±1.1^∗∗∗^	134.8 ± 1.9	119.6±1.3^∗∗∗^	136.7 ± 1.7	119.7±1.9^∗∗∗^
Diastolic blood pressure (mmHg)^a^	79.1 ± 0.7	76.9 ± 0.6^∗^	79.3 ± 1.3	77.8 ± 0.8	79.0 ± 0.9	76.1 ± 1.1^∗^
Creatinine (mg/dl)	0.8 ± 0.01	1.1±0.02^∗∗∗^	0.9 ± 0.04	1.2±0.03^∗∗∗^	0.8 ± 0.01	1.0±0.03^∗∗∗^
Cholesterol (mg/dl)^a^	163.3 ± 1.5	164.3 ± 2.8	159.8 ± 3.2	163.8 ± 3.7	164.5 ± 1.6	165.2 ± 4.0
HDL (mg/dl)^a^	38.6 ± 0.4	37.8 ± 0.6	36.9 ± 0.9	38.1 ± 0.8	39.1 ± 0.5	37.3 ± 1.0
LDL (mg/dl)^a^	94.9 ± 1.2	95.1 ± 2.2	93.1 ± 3.0	94.6 ± 3.0	95.5 ± 1.3	95.9 ± 3.1
Triglycerides (mg/dl)^a^	151.9 ± 2.6	160.5 ± 5.9	150.2 ± 5.6	157.9 ± 7.3	152.5 ± 2.9	165.2 ± 10.0
ALT (U/l)	44.8 ± 0.8	59.8±3.7^∗∗∗^	44.7 ± 1.3	60.4 ± 4.3^∗^	44.9 ± 1.0	58.8±6.9^∗∗^
FPG (mmol/l)	4.4 ± 0.05	5.2±0.1^∗∗∗^	4.4 ± 0.1	5.0 ± 0.2^∗^	4.4 ± 0.1	5.5±0.2^∗∗∗^

The IHD patients were recruited from the patients admitted in the National Institute of Cardiovascular Diseases Hospital (NICVDH), Dhaka, Bangladesh. The control participants were recruited from a suburb of Dhaka, Bangladesh. All participants were on overnight (10 h) fasting and investigated for all the physical and biochemical tests described above. *Statistics*: values are expressed as mean ± SEM (values have been shown as median (range) in Table 2). Statistical significance of the difference between two respective groups was tested using the unpaired two-tailed Student's *t*-test. *p* < 0.05 is considered significant. ^∗^*p* < 0.05. ^∗∗^*p* < 0.01. ^∗∗∗^*p* < 0.001. ^a^The value might be influenced because most IHD patients were on antihypertension and antidyslipidemia (anticholesterol) medications.

**Table 2:** Characteristics of healthy control participants and ischemic heart disease (IHD) patients.

Characteristics	Total participants		Males		Females	
	Healthy	IHD	Healthy	IHD	Healthy	IHD
No. of participants	331	292	84	187	247	105
Age of participants (yr.)	50.0 (35.0-70.0)	52.0 (35.0-70.0)^∗^	52.0 (35.0-70.0)	55.0 (35.0-70.0)	50.0 (35.0-70.0)	50.0 (35.0-70.0)
Weight (kg)	58.0 (34.0-100.0)	60.0 (38.0-115.0)^∗∗^	64.5 (38.0-93.0)	62.0 (38.0-115.0)	55.0 (34.0-100.0)	56.0 (38.0-80.0)
Height (m)	1.5 (1.2-1.8)	1.6 (1.2-1.8)^∗∗∗^	1.6 (1.4-1.8)	1.7 (1.3-1.8)^∗^	1.5 (1.2-1.7)	1.5 (1.2-1.7)
BMI (kg/m^2^)	25.0 (18.5-46.1)	23.5 (16.4-44.4)	24.6 (18.8-33.1)	23.1 (16.4-42.2)	25.3 (18.5-46.1)	25.5 (17.7-44.4)
Systolic blood pressure (mmHg)	133.0 (87.0-229.0)	120.0 (80.0-182.0)^∗∗∗^	131.5 (101.0-193.0)	120.0 (80.0-160.0)^∗∗∗^	133.0 (87.0-229.0)	120.0 (90.0-182.0)^∗∗∗^
Diastolic blood pressure (mmHg)	77.0 (49.0-132.0)	80.0 (50.0-110.0)	78.0 (57.0-125.0)	80.0 (60.0-110.0)	77.0 (49.0-132.0)	79.0 (50.0-110.0)
Creatinine (mg/dl)	0.8 (0.4-2.8)	1.1 (0.5-4.8)^∗∗∗^	0.9 (0.5-2.8)	1.2 (0.7-4.8)^∗∗∗^	0.8 (0.4-1.3)	0.9 (0.5-2.6)^∗∗∗^
Cholesterol (mg/dl)	162.0 (84.3-292.0)	160.0 (65.0-456.0)	156.0 (94.0-280.0)	160.0 (65.0-456.0)	162.0 (84.3-292.0)	162.0 (94.0-298.0)
HDL (mg/dl)	40.0 (21.0-87.6)	38.0 (12.0-58.0)	40.0 (21.0-55.0)	38.0 (12.0-58.0)	40.0 (21.0-87.6)	36.0 (19.0-56.0)
LDL (mg/dl)	92.6 (27.8-188.6)	92.6 (13.0-352.4)	88.0 (43.6-188.6)	92.1 (13.0-352.4)	93.8 (27.8-177.0)	96.0 (17.00-211.2)
Triglycerides (mg/dl)	153.0 (62.0-405.0)	135.5 (17.0-950.0)^∗^	145.0 (63.0-305.0)	134.0 (17.0-950.0)	154.0 (62.0-405.0)	138.0 (27.0-564.0)
ALT (U/l)	44.0 (15.0-215.0)	43.0 (3.0-582.0)	44.0 (15.0-90.0)	44.0 (3.0-548.0)	44.0 (19.9-215.0)	41.5 (5. 0-582.0)
FPG (mmol/l)	4.3 (3.0-7.0)	4.6 (2.5-17.1)^∗∗^	4.5 (3.0-6.2)	4.4 (2.5-17.1)	4.2 (3.0-7.0)	5.0 (2.8-15.5)^∗∗∗^

The recruitment information on participants has been provided in Table 1. *Statistics*: values are expressed as median (range) (values have been shown as mean ± SEM in Table 1). Statistical significance of the difference between two respective groups was tested using the Wilcoxon two independent sample test (Mann–Whitney *U* test). *p* < 0.05 is considered significant. ^∗^*p* < 0.05. ^∗∗^*p* < 0.01. ^∗∗∗^*p* < 0.001.

**Table 3:** Pearson correlation coefficients between IAP level and different risk factors of IHD.

Risk factors	ALL participants		Male		Female	
	Healthy	IHD	Healthy	IHD	Healthy	IHD
Age (yr.)	-0.04410	-0.05934	-0.12718	-0.14064	-0.01674	0.08704
BMI (kg/m^2^)	0.02702	0.06395	0.02645	0.08671	0.01534	-0.01727
Creatinine (mg/dl)	-0.06096	-0.02564	-0.00261	-0.04936	-0.07011	0.10493
Cholesterol (mg/dl)	0.06597	0.09868	0.23047	0.19041	0.01560	-0.05080
HDL (mg/dl)	0.04829	-0.01037	0.07642	0.06194	0.02876	-0.09932
LDL (mg/dl)	0.05118	0.12686	0.22757	0.22708	-0.00577	-0.04219
Triglycerides (mg/dl)	-0.01762	0.01630	-0.01846	0.00957	-0.02013	0.01823
ALT (U/l)	-0.01128	-0.07363	0.32882	-0.00870	-0.07092	-0.14916
FPG (mmol/l)	0.06829	-0.02771	-0.07561	-0.05947	0.09646	-0.00181
Systolic blood pressure(mmHg)	0.00781	-0.01646	0.18112	-0.11468	-0.02604	0.09373
Diastolic blood pressure(mmHg)	0.03910	0.03394	-0.04839	-0.06076	0.05841	0.16490

A Pearson correlation coefficient close to +1 or -1 indicates that the two variables are highly correlated (positively or negatively, respectively). A correlation coefficient between 0 and +0.35 or between 0 and -0.35 was considered of having no correlation between the two variables. *Statistics*: Pearson coefficient was calculated using the SAS program.

**Table 4:** A generalized linear regression model predicts an association of intestinal alkaline phosphatase (IAP) with ischemic heart disease (IHD).

Source	DF	Type III SS	*F* value	Pr > *F*
IHD	1	24854.96	8.59	0.0035
Sex	1	9371.37	3.24	0.0724
Age (yr.)	1	162.05	0.06	0.8130
BMI (kg/m^2^)	1	11.87	0.00	0.9490
Creatinine (mg/dl)	1	7.38	0.00	0.9597
Cholesterol (mg/dl)	1	1536.68	0.53	0.4664
HDL (mg/dl)	1	1770.68	0.61	0.4343
LDL (mg/dl)	1	20.75	0.01	0.9325
Triglycerides (mg/dl)	1	410.99	0.14	0.7064
ALT (U/l)	1	1127.98	0.39	0.5326
FPG (mmol/l)	1	148.22	0.05	0.8210
Systolic blood pressure (mmHg)	1	3602.53	1.25	0.2649
Diastolic blood pressure (mmHg)	1	5263.90	1.82	0.1779

The model predicts a strong association of IAP with IHD. *Statistics*: the SAS program was used for generalized linear modeling (GLM) for regression analysis.

**Table 5:** Multiple logistic regression analysis predicts an association of intestinal alkaline phosphatase (IAP) deficiency with ischemic heart disease (IHD).

Explanatory variables	Logistic coefficient per unit change	Odds ratio (95% CI)
IAP (U/g stool)	-0.00730^∗∗^	0.993 (0.987-0.998)
Sex (ref = male)	-0.3759^∗∗^	0.471 (0.288-0.771)
Age (yr.)	0.0284^∗^	1.029 (1.001-1.057)
Body mass index (BMI, kg/m^2^)	-0.00894	0.991 (0.936-1.050)
Creatinine (mmol/l)	0.0364^∗∗∗^	1.037 (1.025-1.049)
Total cholesterol (mg/dl)	0.00114	1.001 (0.983-1.020)
HDL-cholesterol (mg/dl)	-0.0362	0.964 (0.930-1.000)
LDL-cholesterol (mg/dl)	0.0127	1.013 (0.994-1.032)
Triglycerides (mg/dl)	0.000809	1.001 (0.997-1.005)
ALT (U/l)	0.0110^∗∗^	1.011 (1.003-1.019)
Systolic blood pressure (mmHg)	-0.0746^∗∗∗^	0.928 (0.911-0.945)
Diastolic blood pressure (mmHg)	0.0660^∗∗∗^	1.068 (1.039-1.098)
FPG (mmol/l)	0.4717^∗∗∗^	1.603 (1.339-1.918)
Area under the curve (AUC) = 0.89		

Multiple logistic regression analysis reveals that the IAP level is negatively associated with log-odds of IHD. For one unit (U/g stool) increase in the IAP level, the odds of developing IHD is decreased by 1% controlling for other risk factors, which indicates that high IAP level is probably protective against IHD. *Statistics*: Proc Logist procedure (SAS) was used for multiple logistic regression analysis determining association between IHD and independent risk factors (explanatory variables) including IAP. *p* < 0.05 is considered significant. ^∗^*p* < 0.05; ^∗∗^*p* < 0.01; ^∗∗∗^*p* < 0.001.

## Data Availability

The data described in the manuscript have not been deposited in any repository. All data are available now to the editors and reviewers on request (after deidentification of participants). After publication/acceptance of the paper, all data will be available to any interested person.

## Abbreviations

AP: Alkaline phosphatase
BMI: Body mass index
CHD: Coronary heart disease
IHD: Ischemic heart disease
FPG: Fasting plasma glucose
IAP: Intestinal alkaline phosphatase
IAP-KO: IAP knockout
LPS: Lipopolysaccharides
LTA: Lipoteichoic acids
T2DM: Type 2 diabetes mellitus
TNAP: Tissue nonspecific alkaline phosphatase.
